# Antioxidant, Antityrosinase and Antitumor Activity Comparison: The Potential Utilization of Fibrous Root Part of *Bletilla striata* (Thunb.) Reichb.f.

**DOI:** 10.1371/journal.pone.0058004

**Published:** 2013-02-28

**Authors:** Fusheng Jiang, Weiping Li, Yanfen Huang, Yitao Chen, Bo Jin, Nipi Chen, Zhishan Ding, Xinghong Ding

**Affiliations:** 1 Institute of Biotechnology, College of Life Science, Zhejiang Chinese Medical University, Hangzhou, China; 2 Department of Medicinal Chemistry, College of Pharmaceutical Science, Zhejiang Chinese Medical University, Hangzhou, China; 3 Analysis and Testing Centre, Zhejiang Chinese Medical University, Hangzhou, China; University of Sassari, Italy

## Abstract

This study was carried out to evaluate the utilization probability of the fibrous root part (FRP) of *Bletilla striata*, which was usually discarded and harvesting pseudobulb part (PSP). The chemical composition, total phenolic content, DPPH radical scavenging activity, Ferric-reducing antioxidant power and tyrosinase inhibition activity were compared between FRP and PSP. Antioxidant and pro-oxidant effect as well as antitumor effect of the extract of FRP and PSP were analyzed by *in vitro* cell system as well. Thin layer chromatography and high performance liquid chromatography analysis indicated that the chemical compositions in the two parts were similar, but the content in FRP was much higher than PSP. Meanwhile, the FRP extracts showed higher phenolic content, stronger DPPH scavenging activity, Ferric-reducing antioxidant capacity and tyrosinase inhibition activity. Sub-fraction analysis revealed that the distribution characteristic of phenolic components and other active constituents in FRP and PSP were consistent, and mainly deposited in chloroform and acetoacetate fractions. Especially, the chloroform sub-fraction (sch) of FRP showed extraordinary DPPH scavenging activity and tyrosinase inhibition activity, with IC_50_ 0.848 mg/L and 4.3 mg/L, respectively. Besides, tyrosinase inhibition activity was even stronger than the positive compound arbutin (31.8 mg/L). Moreover, *In vitro* cell system analysis confirmed that FRP extract exerts comparable activity with PSP, especially, the sub-fraction sch of FRP showed better antioxidant activity at low dosage and stronger per-oxidant activity at high dosage, and both sch of FRP and PSP can dose-dependent induce HepG2 cells apoptosis, which implied tumor therapeutic effect. Considering that an additional 0.3 kg FRP would be obtained when producing 1.0 kg PSP, our work demonstrated that FRP is very potential to be used together with PSP.

## Introduction

*Bletilla striata* (Thunb.) Reichb.f. is a well-known traditional Chinese herb, which was first described in Shennong BenCao Jing (*Shennong's Materia Medica*) 2000 years ago. The medicinal part of *Bletilla* is remedy for many diseases. It reduces the edema of the lung, as well as enhances the hemostasis in the lungs, stomach and nose. When applied topically, *Bletilla* is also used to treat skin cracks, abscesses, burns and freckles when combined with other traditional Chinese medicines. *Bletilla* is used to instigate euphoria, purification of the blood, and the strengthening and consolidation of lungs as described in traditional medicine literatures. *Bletilla* is also used to treat swollen tissues induced by malignant tumors, e.g. breast cancer [1]. In industry, *Bletilla* extract is used as a coating agent and cosmetic additive [2]. In traditional Chinese medicine, *Bletilla* can be added in medicated diets or drinks when stewed together with chicken or duck, extracted by boiling water, or brewed as wine material [2]. The plant also has very high decorative value [2].

With modern technologies of drug analysis, numerous novel structures and compounds were identified in *Bletilla*, such as benzyls, bibenzyls, phenanthrenes, biphenanthrenes, dihydrophenanthrenes, anthracene, phenolic acid and their derivatives (most of which are phenol components) [3]. *Bletilla* is also rich in polysaccharides [4]. Purified polysaccharides from *Bletilla* induced significant proliferation of human umbilical vascular endothelial cells (HUVEC) [5]. The effect was associated with the increased VEGF expression when polysaccharide was added into the HUVEC culture media. Hydrogel prepared from the polysaccharide improved the wound healing on a full-thickness trauma mouse model [6], through attenuation of inflammatory cells infiltration and promotion of cell growth. The inhibition of the tumor necrosis factor alpha (TNF-α) level and the elevation of the epidermal growth factor (EGF) secretion were observed after administrating the Hydrogel [6]. Takagi *et al*. [7] also found that *Bletilla* can promote the regeneration of the wound tissue through its anti-infection effect. Among the five antibacterial compounds isolated from the ethyl acetate extract of *Bletilla*, two of them are dihydrophenanthrenes and three of them are bibenzyls [7]. The compounds from *Bletilla* also show anti-tumor effect. Eight stilbenoids isolated from the tubers of *Bletilla* were screened by Morita *et al.* [8]. They confirmed that the anti-mitotic effect in two of the compounds (3,3′- dihydroxy-2′,6′-bis(p-hydroxybenzyl)-5-methoxybibenzyl and 3′,5-dihydroxy- 2-(p-hydroxybenzyl)-3-methoxybibenzyl), through inhibition of tubulin polymerization at IC_50_ 10 µM. Furthermore, the former compound reversed the resistance to chemotherapy agent SN-38 mediated by breast cancer resistance protein (BCRP). In recent years, accumulating evidence shows that the ethanolic extract of *Bletilla* has hypopigmenting activity both in cell-free system [9] and *in vitro* mouse melanoma cell model [10]. Applications of *Bletilla* in tumor therapy and cosmetics are the additional reasons for the extensively exploitation *Bletilla* in recent years.

In China, the natural resources of *Bletilla* have been severely damaged because of the destructive herborization driven by the high demands [2]. The price of *Bletilla* has soared 20-fold in the past 10 years [2]. As part of the efforts to protect the precious plant and explore the best way to utilize the entire plant, our laboratory established good agricultural practice for the growth of *Bletilla* from 2009 onwards. The traditional harvest of *Bletilla* is to collect just the medicinal pseudobulb part (PSP) and discard the fibrous roots part (FRP). We noticed that the fibrous roots and the pseudobulb are structurally interwoven and are difficult to be completely separated. However, there have been no studies investigating whether the FRP of *Bletilla* contains medicinal components, though it is known that different parts of the plant contain similar components. They can be used for remedies if prepared properly [11], [12]. In certain cases, higher yield of active compounds could be found in non-medicinal parts. For example, the total ginsenoside in the fibers of wild ginseng is almost five-fold of the content in root [13]. The Rg_1_ and Rb_1_ contents in fibers of notoginseng also meet the criteria of the Chinese Pharmacopoeia. Therefore, the fibers can be used together with the root of notoginseng as medicine [14]. In current study, we hypothesized that the fibrous roots of *Bletilla* might have similar effective components as pseudobulb. We analyzed fibrous roots and pseudobulb of the *Bletilla* with respect to the total phenolic content, DPPH radical scavenging assay, antioxidant activity and tyrosinase inhibitory activity. Our study suggested that the fibrous roots of *Bletilla* were very valuable and potentially could be used together with pseudobulb.

## Materials and Methods

### Ethics Statement

No specific permits were required for the described field study. The study is not privately-owned or protected in any way. The field studies did not involve endangered or protected species.

### Materials

The principle reagents used, and their sources were as follow: 1,1-diphenyl-2-picrylhydrazyl (DPPH) was purchased from Wako Pure Chemical Industries Ltd. Tyrosinase was purchased from HeFei BoMei Biotechnology Co. Ltd (1680 U/mg), L-Dopa was purchased from Sigma-Aldrich (St Louis, MO, USA). HPLC grade acetonitrile and acetic acid were purchased from Fluka Chemie, water for the mobile phase was twice distilled. Human hepatocellular carcinoma cell line HepG2 purchased from the Cell Bank of The Chinese Academy of Science (Shanghai, P.R. China). RPMI-1640 was purchased from Gibco/invitrogen Corp., Carlsbad, CA, USA. 2′,7′-dichlorodihydrofluorescein diacetate (DCFH-DA) was purchased from Sigma-Aldrich, Shanghai, Trading Co,.Ltd. Trichloroacetic acid (TCA), potassium phosphate, trichloride ferric (FeCl_3_), potassium ferricyanide and other chemicals and reagents were analytical grade and were purchased from Hangzhou Hede Chemical Co. Ltd.

### Plant materials

Three-year-old *Bletilla striata* (Thunb.) Reichb.f. grown at Jiangshan city (Zhejiang, China) were collected in October 2011, and were authenticated by Professor Yao Zhensheng (Zhejiang Chinese Medical University, China). The fibrous roots and pseudobulb were separated, air-dried and powdered respectively, then stored in desiccator at 4°C for further use.

### Extraction of Bletilla striata

The powdered fibrous roots part (FRP) and pseudobulb part (PSP) of *Bletilla* were extracted with 95% ethanol and then with distilled water under reflux. The filtered extracts were vacuum-dried in a rotary evaporator at 40°C, and stored at 4°C for further analysis. In addition, the partial 95% ethanol extraction of both PSP and FRP was concentrated and diluted with distilled water, then sequentially fractionated into petroleum (spe), chloroform (sch), acetoacetate (sac), n-butanol (sbu) and water (swa) five sub-fractions, respectively.

### TLC analysis

The constituents of the PSP and FRP were compared by the thin-layer chromatography (TCL). Samples were dissolved in suitable solvent at final concentration of 20 mg/ml. 10 µl of each samples was spotted on silica gel GF254 plate (normal phase, Branch of Qingdao Haiyang Chemical Co. Ltd. Shandong, China), and developed with the following system: chloroform/methanol/water (6/0.5/0.05, v/v/v) [15]. The plate was air dried and recorded under UV light, then developed with I_2_/KI under room temperature, and 5% H_2_SO_4_-ethanol in 110°C for five minutes sequentially. Results were photographed and analyzed.

### HPLC analysis

For HPLC analysis, 0.5 g powder of FRP and PSP were precisely weighted and refluxed with 50 ml 95% ethanol for 1.5 h, respectively. Filtrate were concentrated and metered volume to 10 ml with 95% ethanol, after filtrate with 0.22 µm filter, 5 µl and 15 µl samples of FRP and PSP were injected and analyzed using a Dionex UltiMate™ 3000 HPLC system with PAD at 260 nm, respectively. A Dionex Acclaim120 C18 (250×4.6 mm, 5 µm) HPLC column protected with a Phenomenex security guard column (C18, 4×3.0 mm) operated at 30°C was used, and the flow rate was maintained at 1 ml/min. The elution solvents were acetonitrile (A) and 0.1% acetic acid (B). Samples were eluted according to the following gradient: 0–25 min 30% A isocratic, 25–35 min 30% to 40% A, 35–45 min 40% A isocratic, 45–70 min 40% to 100% A, and finally washing and recondition of the column. Identification of peaks was achieved by comparing retention times and their UV-VIS spectra from 190 to 400 nm. Each sample was measured in triplicate, and the peak area was compare between FRP and PSP.

### Measurement of total phenol content

The total soluble phenolic content in the FRP and PSP of *Bletilla* were determined with the Folin-Ciocalteu reagent according to the method of Slinkard and Singleton [16] with slight modifications. Briefly, samples were made up to the final volume (2.0 ml) with methanol, and then thoroughly mixed with 1.0 ml Folin-Ciocalteu reagent at 25°C. Ten minutes later, 2.0 ml of 1.0 M Na_2_CO_3_ was added, followed by mixing with intermittent shaking and incubation at 50°C for 10 min. Absorbance at 770 nm was read by the spectrophotometer. Each sample was measured in triplicate, and the data were expressed as gallic acid equivalent (GAE) per mg dry weight of the sample, based on the standard curve of gallic acid (R = 0.9996).

### DPPH radical scavenging assay

The free radical scavenging activity of FRP and PSP of *Bletilla* were measured using the methods of Marxen *et al.* [17] with slight modification. Initially, 2.0 ml of methanol solution containing 0.2 ml of each of the samples at different concentrations was mixed with 0.5 ml of DPPH stock solution (The final concentration of DPPH is 76 µM, Wako Pure Chemical Industries Ltd, Osaka, Japan). The mixture was then incubated for 30 min at room temperature. The control group contained all reagents without the sample, whereas methanol was used as a blank. All measurements were performed in triplicate. DPPH radical scavenging activity was calculated according to the formula: Scavenging activity (%)  = 
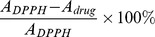
. Where, *A*_DPPH_ and *A*_drug_ was the absorbance of control and tested sample respectively.

### Ferric-reducing antioxidant power (FRAP) assay

Ferric-reducing antioxidant power of *Bletilla* samples were measured using the assay described by Wazir *et al.* [18]. About 0.5 ml of potassium phosphate buffer (0.2 M, pH 6.6) and 0.5 ml 1% potassium ferricyanide was thoroughly mixed with 0.3 ml *Bletilla striata* extracts. The mixture was incubated at 50°C for 20 min. The reaction was stopped with 0.5 ml of 10% trichloroacetic acid (TCA), and then centrifuged for 5 min at 3 000 g. 1.0 ml of the supernatant was mixed with as equal volume of distilled water, followed by adding 0.1 ml of 0.1% ferric chlorate (FeCl_3_). The absorbance was measured at 700 nm. Gallic acid was the positive control while methanol was the negative control. All the measurements were in triplicate. Reducing power was determined from the plot of optical density against concentration of extract, and reducing power (RP 0.5_AU_) was the concentration of extract that rose 0.5 of the absorbance.

### Tyrosinase inhibition assay

Tyrosinase activity assay was performed in 96-well microplate with L-DOPA as the substrate [19]. Briefly, four groups were designated as A, B, C and D. They contained the following reaction mixtures: group A, tyrosinase (11.5 units/ml, working concentration, purchased from HeFei BoMei Biotechnology Co. Ltd, Anhui, China, 1680 U/mg) only; group B, 160 µl 1/15 M phosphate buffer (pH 6.8) as blank; group C, tyrosinase (11.5 units/ml) + sample solution; group D, sample solution only. The contents of each well were mixed completely and then incubated at 25°C for 10 min, followed by the adding of 40 µl of 2.5 mM of L-DOPA. After incubation at 25°C for 10 min, the absorbance at 475 nm of each well was measured. The percentage inhibition of the enzyme by the active extracts was calculated as following: Tyrosinase inhibition activity %  = 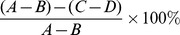. The inhibitory effect (%) of the compound was expressed as the inhibitor concentration causing 50 % loss of enzyme activity (IC_50_). All the measurements were performed at least in triplicate, and arbutin (Sigma Chemical Co. St. Louis, U.S.A) was adopted as positive control.

### Measurements of intracellular ROS

Intracellular ROS levels were determined by using the ROS molecular probe 2′,7′-dichlorodihydrofluorescein diacetate (DCFH-DA) (Sigma-Aldrich, Shanghai, Trading Co,.Ltd) as previously described with minor modification [20]. HepG2 cells were seeded into 96-well tissue culture plate at a density of 2.5×10^4^ cells/well, cultured in RPMI1640 medium (Gibco/Invitrogen Corp., Carlsbad, CA, USA) supplemented with 10% heat-inactivated FBS, 100 U/ml penicillin/streptomycin (Sigma, St. Louis, MO, USA) under a humidified atmosphere containing 5% CO_2_ at 37°C and allow to stabilize for 24 hours. Then cells were washed once with warm D-PBS buffer and incubated with a 10 µM ROS-sensitive fluorescent probe DCFH-DA for 30 min at 37°C. Washed away the free probe and the cells were treated with different concentrations of samples for 1.5 h at 37°C. After treatments, cells were washed twice with cold PBS, each well was filled with 150 µl of cold PBS and cell suspensions were sonicated. The resulting cell homogenates were applied to measure fluorescence intensity using a Thermo-Labsystems Varioskan Flash Multimode Spectral Scanning Microplate Reader (Thermo Scientific Co.). Excitation and emission wavelengths used for fluorescence quantification were 488 and 530 nm, respectively. Total protein concentration of each sample was determined using CB-protein assay kit (Merck Biosciences. co.). All fluorescence measurements were adjusted for background fluorescence and protein concentration. Using untreated cells as reference, the antioxidant and pro-oxidant outcome was evaluated by comparison of three measurements and expressed as a percentage of untreated controls.

### Cell viability assay

To determine cell viability the colorimetric (3-(4,5-Dimethylthiazol-2-yl)-2,5- diphenyltetrazolium bromide (MTT, Sigma-Aldrich Co.) metabolic activity assay was used as previously described [21]. HepG2 cells were grown in RPMI1640 medium with 10% FBS and 100 U/ml penicillin/streptomycin were seeded into 96-well tissue culture plate at a density of 1×10^4^ cells/well at 37°C, and exposed to varying concentrations of samples for 24 h. Cells treated with medium served as a negative control. At the end of the treatment, 20 µl of fresh serum-free medium containing MTT (5 mg/ml) was added into each well. Cells were incubated for another 4 h, the resulted formazan crystals were dissolved in dimethyl sulfoxide (100 µl) and the respective absorbance intensity was measured by a Varioskan Microplate Reader (Thermo scientific Co.) at 570 nm with a reference wavelength of 620 nm. All experiments were performed in triplicate, and the relative cell viability (%) was expressed as a percentage relative to the untreated control cells.

### Apoptosis assay using flow cytometric and microscopic method

Flow cytometric evaluation of Annexin V-FITC/propridium iodide (PI) staining was used to determine the predominant form of cell death induced by drug treatment. Intact (normal) cells (FITC−/PI−), apoptotic cells (FITC+/PI−) and necrotic cells (FITC+/PI+) were quantified by flow cytometry using the Annexin V-FITC Apoptosis Detection Kit I (PharMingen, San Diego, CA). In brief, following sample treatment of HepG2 cells for 24 h at various concentrations, cells were harvested and pelleted by centrifuging at 1,000 g for 5 min at room temperature. Cells were washed twice with cold PBS and resuspended in binding buffer at a concentration of 2×10^6^ cells/ml. Annexin V-FITC (5 µl) and PI (10 µl) were added to 100 µl of cells. The tube was vortexed gently and incubated in the dark for 15 min at room temperature. Additional 400 µl binding buffer was added to each tube and samples were analyzed by FACScan using Cell Quest software (both from Becton-Dickinson, San Jose, CA).

To verify the results of flow cytometry assays, fluorescence microscopy was performed after staining cells with Hoechst33342 (5 µg/ml) and PI (10 µg/ml). Cells were seeded into 24-well plate at a density of 4×10^4^ cells/well and cultured at 37°C, 5% CO_2_ incubator for 24 h, and exposed to different samples or vehicle for another 24 h. At the end of the treatment, cells were washed twice with cold PBS, exposed for 20 min in the dark to Hoechst33342 and PI, and examined by fluorescence microscopy (Olympus Imaging China Co., Ltd.).

### Statistical analysis

Results are presented as mean values ± standard deviation. Analysis of variance and significant differences among means were determined by one-way ANOVA using SPSS (Version 13, SPSS Inc., Chicago, USA.). Significant differences were declared at *P*<0.05.

## Results and Discussion

### Production and extraction content from PSP and FRP

**Figure 1:**
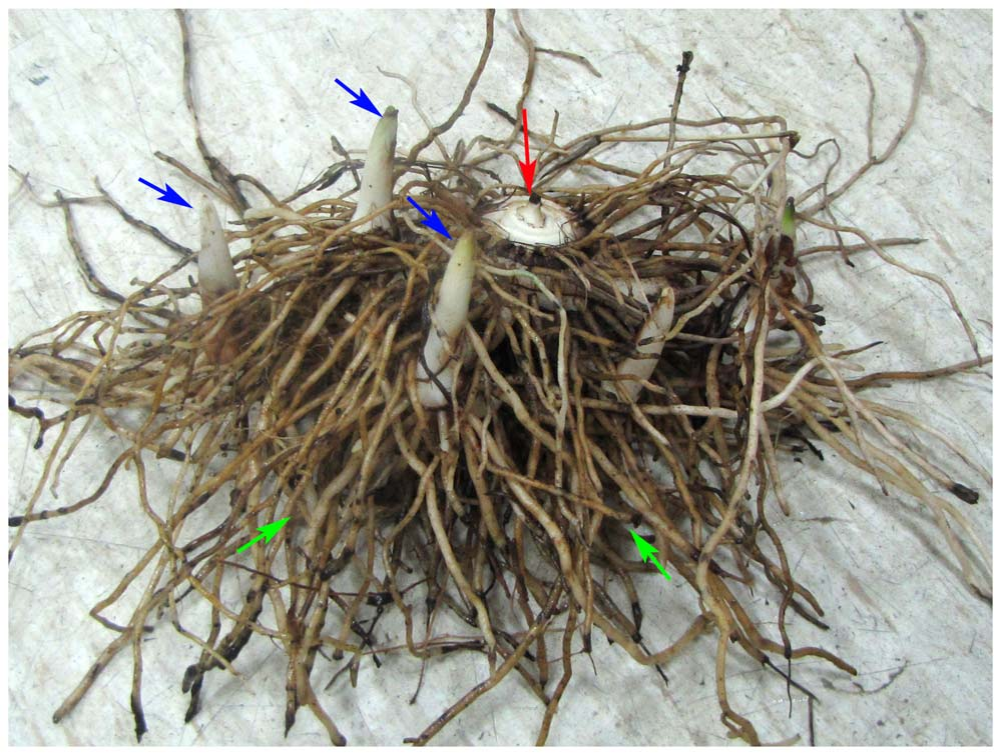
The underground part of a three-year-old *Bletilla* plant. Red, green and blue arrows indicate pseudobulb, fibrous roots and shoots of *Bletilla* respectively.

Six batches of *Bletilla* samples from different experimental fields were collected randomly. After clearing off the soil, PSP and FRP were separated from the underground part (Fig. 1). The ratio of PSP/FRP for fresh or dry weight was 3.34±0.23 or 3.75±0.34. The ratio of fresh/dry weight for PSP or FRP was 4.04±0.35 or 3.65±0.44, respectively. Furthermore, the ethanolic extract content between PSP and FRP which approximately 11–15% of the dry weight were not significantly different. The aqueous soluble substance was more than 30% and 20% of the dry weight of PSP and FRP, respectively. These data demonstrated the potentially medicinal value of the FRP, which was traditionally discarded as waste, with an estimated 30% of the total weight of *Bletilla* plant.

### Thin layer chromatography (TLC) analysis

**Figure 2:**
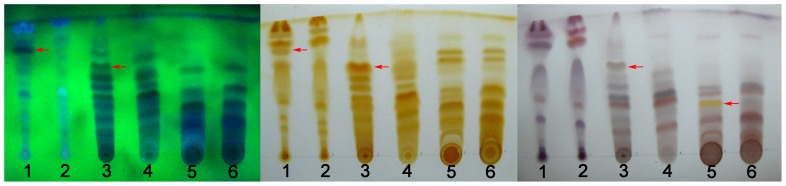
Analysis of the constituents of the PSP and FRP by TLC method. The plate was air dried and recorded under UV light (panel A), then developed with I_2_/KI under room temperature (panel B), and 5% H_2_SO_4_-ethanol in 110°C for five minutes (panel C) sequentially. The lane 1, 3 and 5 were loaded with the sub-fractions of spe, sch and sac from the FRP; lane 2, 4 and 6 were loaded with the sub-fractions of spe, sch and sac from the PSP. Red arrows indicated the components that are absent in the PSP.

There are too many constituents in the 95% ethanol extract, and some low content compounds were difficult to be manifested on the plate. Three subfractions, spe, sch and sac, were spotted and developed for comparison. As demonstrated in Fig. 2, almost all the constituents in PSP were also detected in FRP, and even more components were discovered in FRP (Fig. 2, red arrows indicated). These results implied that FRP would exert similar therapy effect as PSP.

### High-performance liquid chromatography (HPLC) analysis

**Figure 3:**
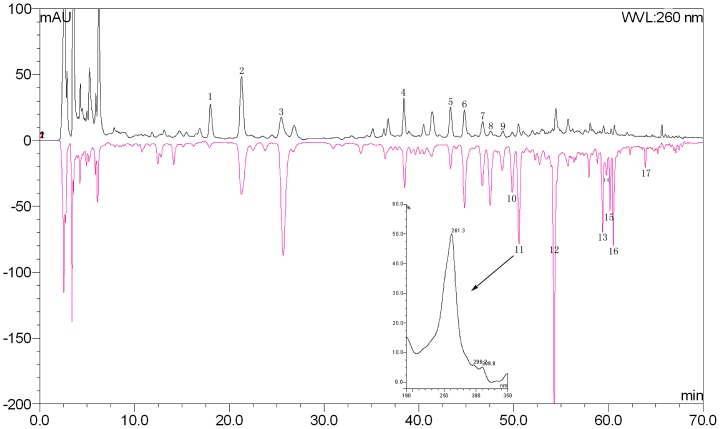
The HPLC results of tubers and fibrous roots of *B. striata*. The black and red curve represent for PSP (15 µL injection) and FRP (5 µL injection) 95% ethanol extract, respectively. Peak marked was identified as the same compound both existed in PSP and FRP by UV-VIS spectrum.

PAD detect results indicated most of the peak with maximal absorption around 260 nm, and with similar UV spectrum as exhibited in Fig. 3. (peak 11), which implied that abundant of analogues composed the chemical constituents of *Bletilla*, and this finding was consistent with those reports [3], [7], [8]. As expected, the chemical composition of FRP was mainly coincidence with PSP, but it was noticeable that the content of the most of the components in FRP were significantly higher than that of PSP (Table 1). We presumed that FRP would have stronger pharmaceutical activity.

**Table 1:** The HPLC peak area comparing of PSP and FRP.

Peak NO.	Retention time (min)	Peak area (mAU*min)		Ratio of area(FRP/PSP)
		FRP	PSP	
1	17.900	1.602±0.184	2.889±0.108	0.555
2	21.283	24.355±0.154	6.416±0.241	3.796
3	25.683	48.542±0.335	3.051±0.098	15.912
4	38.425	10.926±0.114	2.362±0.088	4.626
5	43.350	5.416±0.015	2.429±0.101	2.229
6	44.817	18.174±0.158	2.401±0.154	7.568
7	46.725	10.936±0.224	1.327±0.124	8.241
8	47.567	17.365±0.178	0.478±0.048	36.354
9	48.850	7.452±0.091	0.469±0.084	15.889
10	49.850	11.035±0.118	0.444±0.058	24.835
11	50.517	20.897±0.317	1.004±0.102	20.807
12	54.467	50.946±0.284	2.413±0.214	21.110
13	59.509	11.964±0.131	0.334±0.101	35.856
14	59.909	5.479±0.189	0.025±0.009	219.160
15	60.292	7.887±0.084	0.162±0.077	48.786
16	60.642	15.189±0.247	0.428±0.058	35.516
17	64.000	0.751±0.104	0.049±0.004	15.223

Note: n = 3, area was calculated as 5 µL injection.

### Total phenolic content

**Figure 4:**
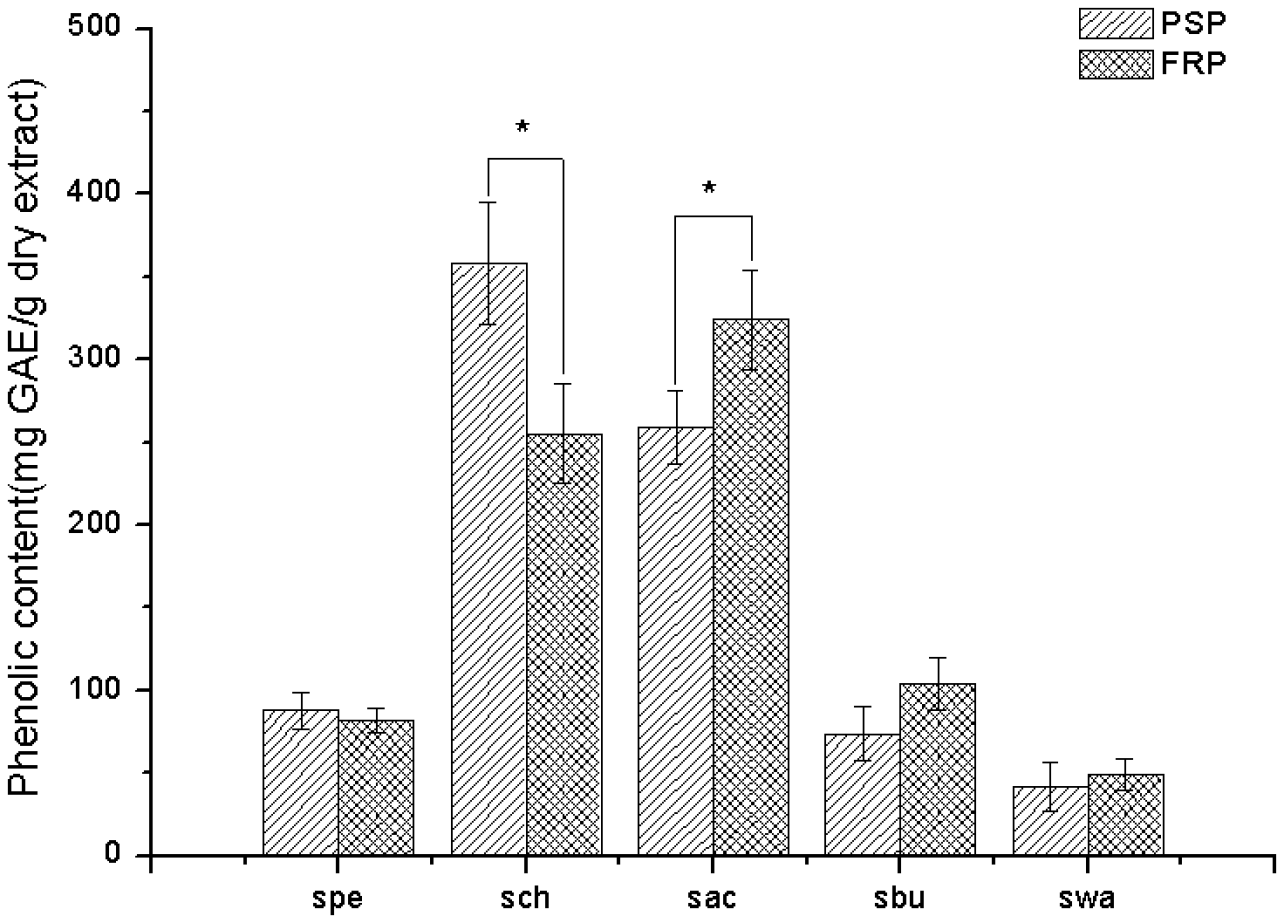
The phenolic content in sub-fractions of the PSP and FRP (*, *P*<0.05, n = 3).

The total phenolic content is much higher in FRP than in PSP (8.95±0.56 and 3.94±0.39 mg GAE/g dry weight, respectively). The content of PSP was comparable to the value reported [22]. Phenolic compounds were considered to be natural antioxidants. In general, there is a good correlation between the free radical scavenging activity (antioxidant activity) and the total phenolic content in the plant [23], [24]. Thus the results suggest that FRP would have better antioxidant activity. Additionally, the phenolic content of the sub-fractions of 95% ethanolic extract were determined as well, as shown in Fig. 4. The distributive patterns of phenolic content among sub-fractions were similar between PSP and FRP. Most of the phenolic content was found in ach and sac, and more than 250 mg GAE/g dry extract. The content from these two sub-fractions may exert great pharmacodynamic activity.

### DPPH Radical-Scavenging Activity

**Figure 5:**
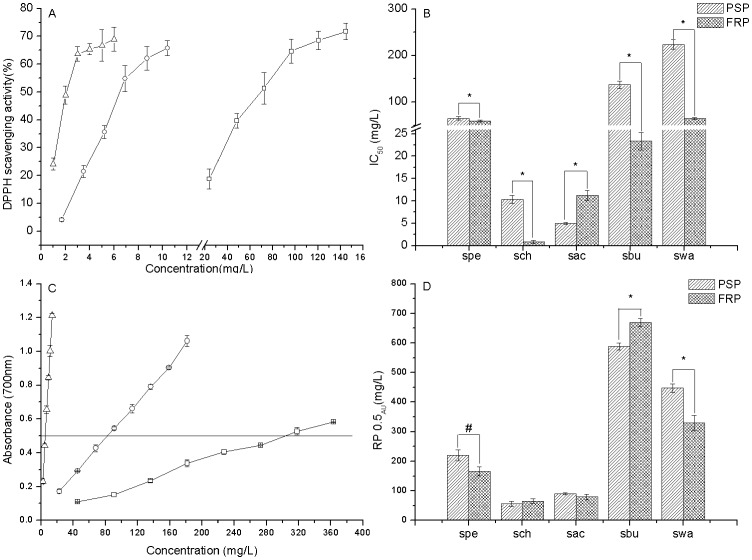
DPPH scavenging activity and Ferric-reducing antioxidant power of the extracts. The symbol □, ○, *Δ* in panel A and C represent for 95% ethanol extract of the PSP, 95% ethanolic extract of the FRP and the positive control of gallic acid, respectively; Panel A and B, DPPH scavenging activity; Panel C and D, Ferric-reducing antioxidant power; ^#^, *P*<0.05; *, *P*<0.01; n = 3.

**Figure 6:**
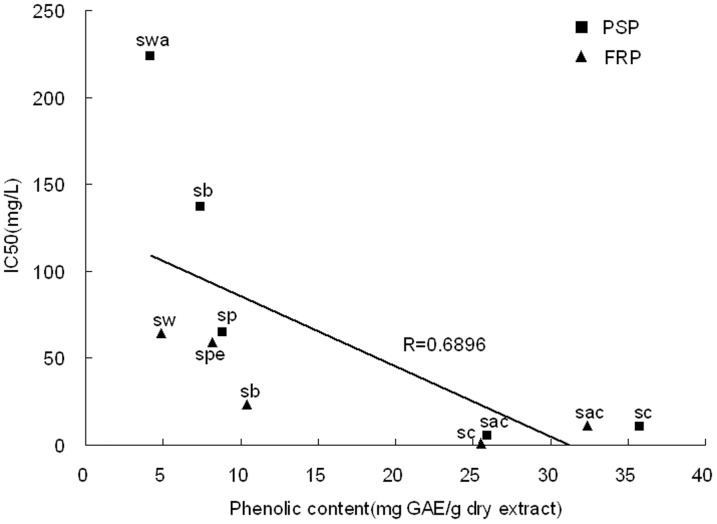
Correlation analysis between the DPPH scavenging activity (IC_50_) and the phenolic content for sub-fractions of the PSP and FRP (*P*<0.05).

The method to measure scavenging activity with DPPH as a stable free radical is widely used to evaluate antioxidant activity in food and plant extracts [25]. The scavenging abilities of extracts against DPPH radical were shown in Fig. 5A. Ethanolic extracts from both FRP and PSP had strong free radical scavenging activity. The IC_50_ of FRP (6.2 mg/L) was slightly lower than the positive control (2.4 mg/L) but was significantly higher than PSP (68.0 mg/L) (Fig. 5A). The IC_50_ values of sub-fractions were shown in Fig. 5B. As expected, the activity was tightly correlated with the content of total phenolic components in the sub-fractions of both PSP and FRP (Fig. 6). Moreover, the FRP extract showed stronger antioxidant activity than the PSP in almost all the sub-fractions except for sac. It is noticeable that sch of FRP manifested the strongest activity with IC_50_ 0.848 mg/L, which was almost 3-fold of the positive control. While ethanolic extract showed strong antioxidant activity, the aqueous extract was absent in any activity. However, Rui *et al.* [26] reported that the neutral polysaccharide isolated from *Bletilla striata* could scavenge ·OH efficiently. The contradictory results might be due to the different free radical models established [27].

### Ferric-reducing antioxidant power assay

FRAP assay is a simple and quick assay that is reproducible and linearly correlated to the activity of antioxidants (e.g. polyphenols) in the plant extracts [28]. In this study, the capacity of the extract from *Bletilla* to reduce iron (III) to iron (II) was determined and compared to gallic acid, which is known for its strong reducing properties. Fig. 4C shows the reductive capacity of ethanolic extract obtained from PSP and FRP linearly related to the sample concentration. It is noteworthy that the reducing power of the ethanolic extract of FRP (RP0.5_AU_ = 83.68 mg/L) is apparently stronger than that of PSP (301.36 mg/L). The reducing power of the sub-fractions (Fig. 5B) parallels to the DPPH radical scavenging activity (Fig. 5D), which is also positively correlated with the phenolic content (see Fig. 6).

### Tyrosinase inhibition activity

Tyrosinase is widely distributed in plants, microorganisms, animals and humans [29]–[31]. It is responsible for skin melanization in animals and humans, and for browning in plants. Tyrosinase inhibitor is used as skin whiting agent and the natural sources of such inhibitors have high market value [32], [33]. *Bletilla striata* is used as skin whitening herb in traditional Chinese medicine. A number of studies have shown that the ethanolic extract from PSP has great tyrosinase inhibitory activity and melanin biosynthesis inhibitory activity both in the cell free system [9], [10]. Lin [34] reported that supercritical carbon dioxide extraction from PSP had better hypopigmenting activity than arbutin, and was similar to kojic acid. Our findings were consistent with those published works, and ethanolic extracts from PSP showed strong tyrosinase inhibition activity in a dose dependent manner with IC_50_ = 751.4 mg/L (Table 2). Surprisedly, we found that the ethanolic extract from FRP showed stronger tyrosinase inhibitory activity with IC_50_ = 359.7 mg/L (Table 2). The aqueous extract from FRP exhibited inhibitory effect to tyrosinase activity, but was lower than the ethanolic extract of FRP and PSP. However, no activity was detected in the aqueous extract of PSP. Further analysis revealed that the majority of the active components were distributed in sch and sac sub-fractions in both of PSP and FRP, and the sch fraction of FRP showed the greatest inhibitory activity, with IC_50_ only 4.3 mg/L, which was much stronger than the positive compound arbutin (31.8 mg/L). The inhibitory activities in all sub-fractions from FRP were significantly more potent than that from PSP. Overall, these data suggest that FRP has higher tyrosinase inhibitory capacity than PSP.

**Table 2:** Tyrosinase inhibitory activity of the extracts of *Bletillae*.

	IC_50_ (mg/L)						
	water extract	95%ethanol extract	spe	Sch	sac	sbu	swa
FRP	850.3±23.5	359.7±35.7^a^	132.5±10.5^a^	4.3±1.5^a^	67.0±8.7^a^	536.0±21.1^a^	518.0±31.0^a^
PSP	—	751.4±51.2	222.1±13.4	29.5±6.8	173.9±14.4	>1000	857.7±44.5
Arbutin	31.8±4.4						

Note: n = 3, a: *p*<0.05, compared with PSP.

### Effect of sub-fractions on HepG2 cells ROS levels

**Figure 7:**
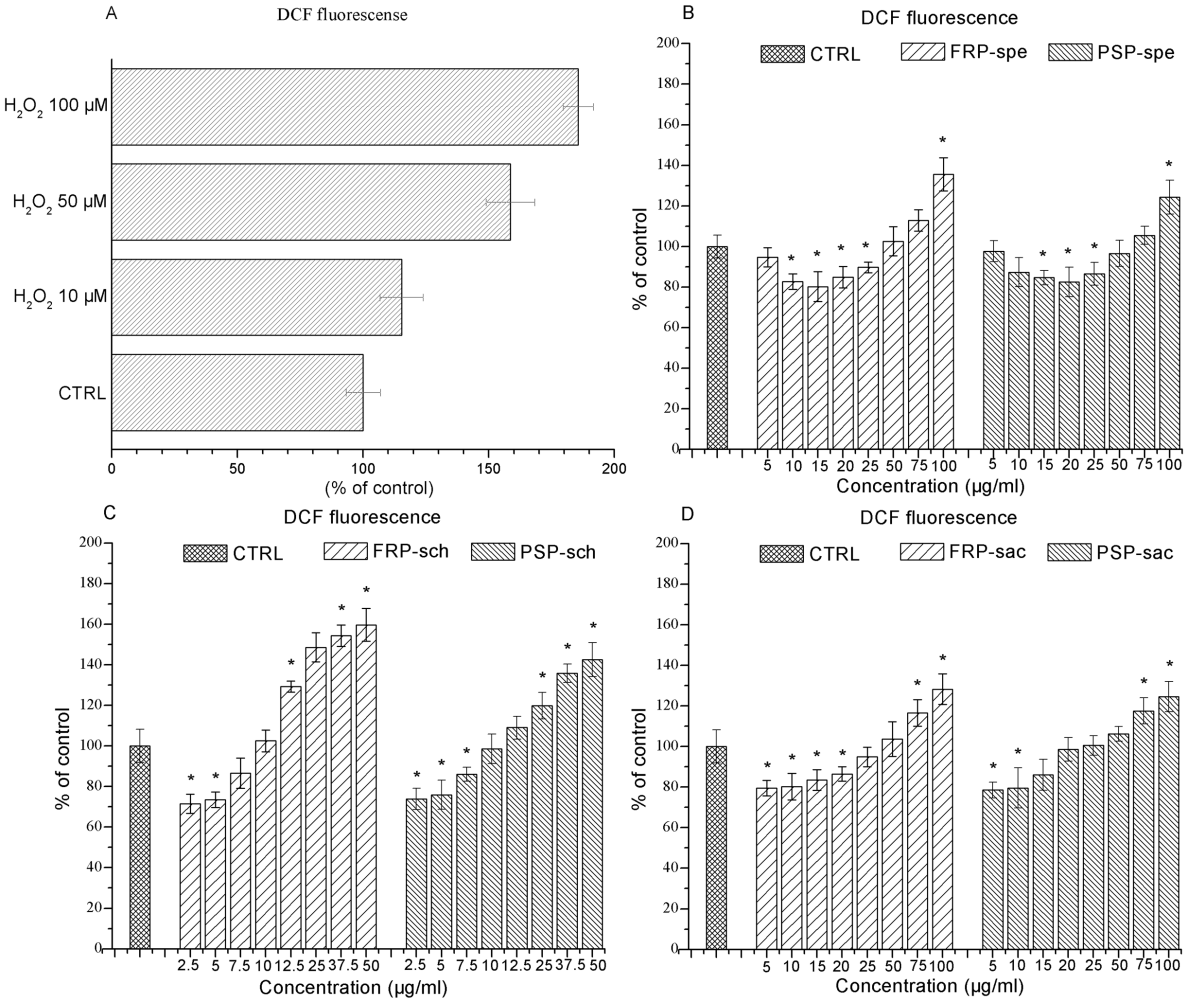
Effect of sub-fractions on intracellular ROS levels in HepG2 cell line. HepG2 cells were stimulated for 1.5 h, intracellular ROS levels were assessed as described in the ‘Materials and Methods’ section. (A) Dose-dependent effect of H_2_O_2_ on intracellular ROS levels; (B,C and D) Intracellular ROS levels in cultured HepG2 in the absence (CTRL) or presence of the indicated concentration of (B) spe, (C) sch and (D) sac sub-fractions of both FRP and PSP. (A–D) * Significantly different from the control, *P*<0.05; n = 3.

To validate the ROS assay, the variations of intracellular ROS levels in response to increasing doses of H_2_O_2_ was detected in HepG2 cell line model. Data in Fig. 7A showed that the fluorescence signal increased in response to H_2_O_2_ (a well-known pro-oxidants) in a dose-dependent manner, and then the effect of the five sub-fractions of both FRP and PSP on the intracellular ROS levels were determined; results were expressed as a percentage of controls. Treatment of HepG2 cells with sub-fractions spe, sch and sac of both FRP and PSP exerted a significant antioxidant effect at low dosage (Fig. 7B, 7C and 7D), which confirmed the protective effect of *Bletilla striata*. Corresponding to the chemical assay system, the sch sub-fractions manifested prominent antioxidant effect below 10 µg/ml. However, the exposure of cell cultures to higher concentrations of spe, sch and sac all increased intracellular ROS levels in dose-dependent manner. These results were distinct to that of previous cell free system. As many publications reported, the antioxidant effect of those sub-fractions (spe, sch and sac) at low dosage was lost and a marked pro-oxidant effect was evident at high dosage [20], [35]; which may imply that compounds impact intracellular ROS levels mainly through compounds-cellular receptor or compounds-cellular signal transductions way, rather than compounds-ROS direct interaction way. Additionally, the sub-fractions sbu and swa for both FRP and PSP showed no significant antioxidant activity.

### Induction of HepG2 impairment and apoptosis by sub-fractions

**Figure 8:**
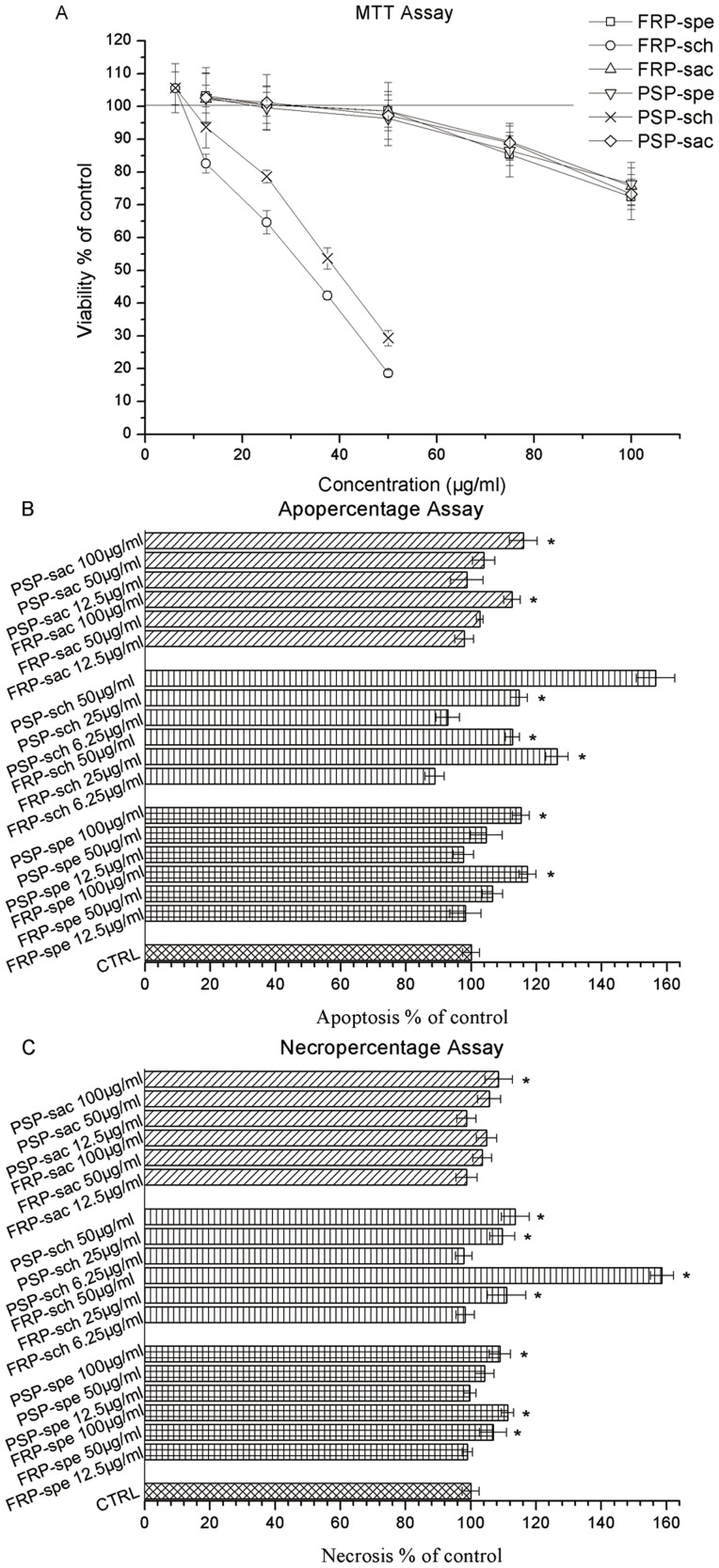
Sub-fractions induce HepG2 impairment and apoptosis. HepG2 cells were exposure in different sub-fractions and concentrations for 24h, and then cell viability and apoptosis were assessed as reported in the ‘Materials and Methods’ section. (A) Cell viability, (B) apoptosis and (C) necrosis in presence of the indicated sub-fraction concentration. Data are expressed as percent of control (CTRL). (A–D) * Significantly different from the control, *P*<0.05; n = 3.

**Figure 9:**
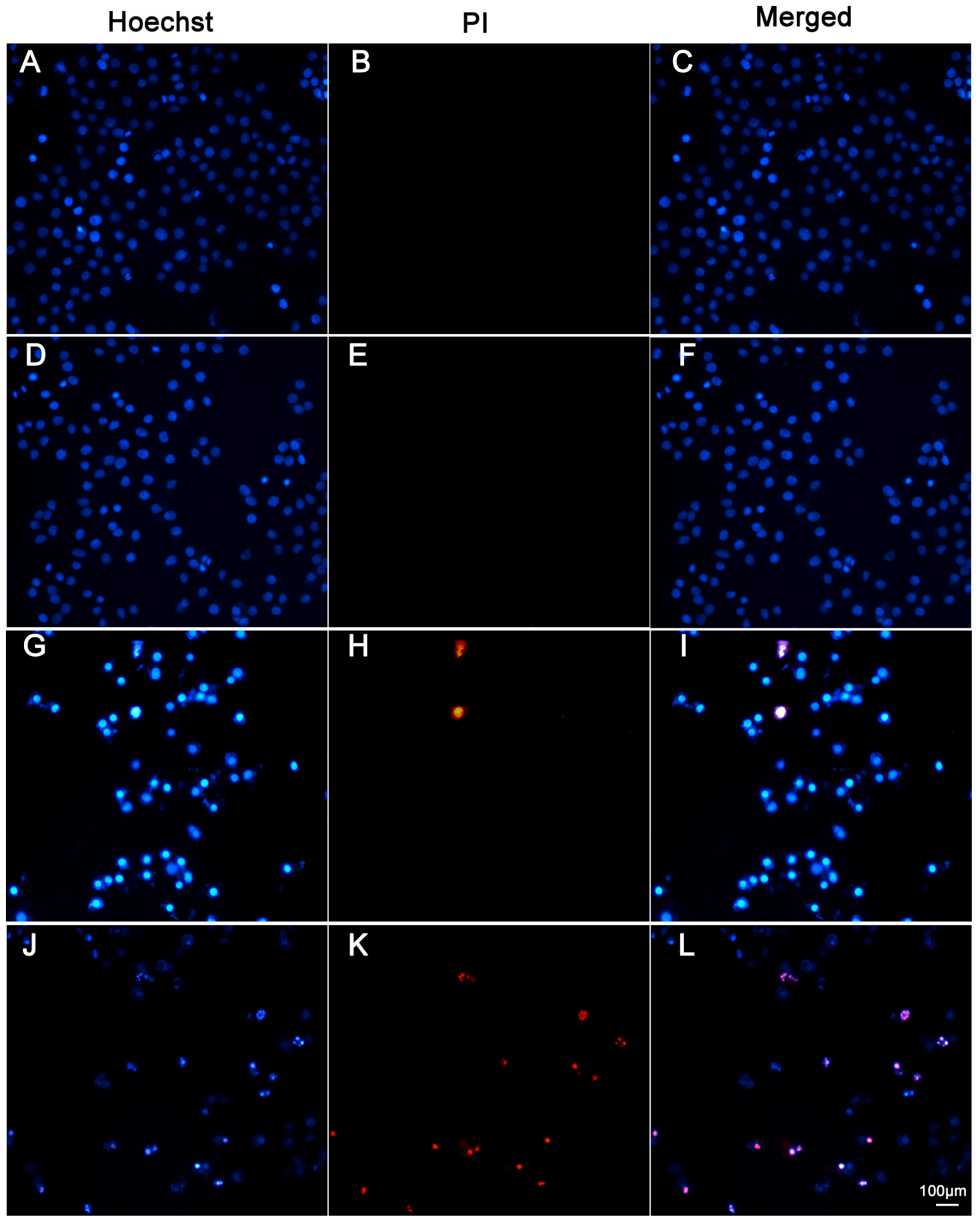
sch-fractions induce HepG2 apoptosis and necrosis. HepG2 cells were treated in different sub-fractions and concentrations for 24 h, and then cell were stained with Hoechst/PI as mentioned in the ‘Materials and Methods’ section. (A–C) Cells were absence of any treatment; (D–F) Cells were exposure to 6.25 µg/ml sch of FRP; (G–I) Cells were exposure to 50 µg/ml sch of PSP; (J–L) Cells were exposure to 50 µg/ml sch of FRP; magnification:×200.

Cell viability was evaluated by MTT assay. Survival rate of HepG2 cells were dose-dependently decreased in response to increasing concentration of sch sub-fractions for both FRP and PSP (Fig. 8A); however, inhibition activity of spe and sac sub-fractions at the dosage below 100 µg/ml was not significant (Fig. 8A), and inhibition activity of sbu and swa sub-fractions was absent. Obviously, the effect of decreased cell survival corresponding to increasing concentrations of sch sub-fractions were consistent with the increase in ROS levels (Fig. 7C). Flow cytometric analysis indicated that the sch sub-fraction of PSP could significantly induce cell apoptosis in a dose-dependent manner (Fig. 8B); however, while viability was decreased dose dependently (Fig. 8A), apoptosis did not increased (Fig. 8B), suggesting a potential shift toward a necrotic mechanism at high sch concentrations of FRP, which was confirmed by our flow cytometric assay (Fig. 8C). Moreover, sch sub-fractions induced cell apoptosis was reconfirmed by microscopic method. Low dosage (6.25 µg/ml) treatment of sch from both FRP (Fig. 9D-9F) and PSP showed no apoptosis or necrosis cells; however, when cells were exposed to 50 µg/ml, the cell numbers were decreased dramatically and the morphology were shrinked significantly (Fig. 9G–9L); increased apoptosis ratio with brighten Hoechst staining was obtained after treatment by sch of PSP (Fig. 9G–9I), while high necrosis ratio with PI staining was observed after treatment by sch of FRP (Fig. 9J–9L), which would be attributed to higher amount of active components from FRP than those from PSP (Fig. 3). Our data support those publications [20], [35] that high dosage antioxidant may exert pro-oxidant effect, and cause tumor cell apoptosis, but its underlining mechanism needs to be uncovered.

## Conclusions

We are the first to report the similarity of the chemical components between FRP and PSP of *Bletillae*. FRP extract has higher total phenolic content, stronger DPPH radical scavenging activity, ferric-reducing antioxidant activity and tyrosinase inhibitory activity. *In vitro* cell system analysis confirms that FRP extract exerts comparable activity with PSP. Especially the sch sub-fraction of FRP shows more significant antioxidant activity at low dosage and per-oxidant activity at high dosage. And the remarkable apoptosis inducing effect of sch sub-fractions on HepG2 cell may imply its anti-tumor therapeutic effect. Moreover, attention should be paid that the IC_50_ of tyrosinase inhibitory activity for sch of PSP and FRP was 29.5 µg/ml and 4.3 µg/ml respectively, and at corresponding dosage, sch of PSP showed low toxicity (Fig. 8A), and sch of FRP exerted antioxidant activity (Fig. 8A and Fig. 7C). Therefore, we conclude that FRP of *Bletillae* has potentially medicinal value as a safe anti-oxidant, whiting agent or antitumor agent.
